# High-dose BEAM chemotherapy with autologous haemopoietic stem cell transplantation for Hodgkin's disease is unlikely to be associated with a major increased risk of secondary MDS/AML

**DOI:** 10.1038/sj.bjc.6690718

**Published:** 1999-10

**Authors:** C N Harrison, W Gregory, G Vaughan Hudson, S Devereux, A H Goldstone, B Hancock, D Winfield, A K MacMillan, P Hoskin, A C Newland, D Milligan, D C Linch

**Affiliations:** University College Medical School, London, UK; Weston Park Hospital, Whitham Rd, Sheffield, UK; Royal Hallamshire Hospital, Glossop Rd, Sheffield, UK; Mount Vernon Hospital, Rickmansworth Road, Northwood, UK; Royal London Hospital, Whitechapel, London, UK; Birmingham Heartands Hospital, Bordesby Green East, Birmingham, UK

**Keywords:** secondary myeloid malignancy, Hodgkin's disease, transplantation

## Abstract

Hodgkin's disease is curable in the majority of patients, although a proportion of patients are resistant to or relapse after initial therapy. High-dose therapy with autologous stem cell support has become the standard salvage therapy for patients failing chemotherapy, but there have been reports of a high incidence of myelodysplasia/acute myeloid leukaemia (MDS/AML) following such treatment. Patients who receive such therapy form a selected group, however, who have already been subjected to other leukaemogenic factors, such as treatment with alkylating agents. In order to ascertain the true risk of MDS/AML, comparison must be made with other patients subjected to the same risks but not undergoing transplantation. We report a retrospective comparative study of 4576 patients with Hodgkin's disease from the BNLI and UCLH Hodgkin's databases, which includes 595 patients who have received a transplant. Statistical analysis including Cox's proportional hazards multivariate regression model with time-dependent covariates was employed. This analysis reveals that the risk of developing MDS/AML was dominated by three factors, namely quantity of prior therapy (relative risk [RR] 2.01, 95% confidence intervals [CI] 1.49–2.71, for each treatment block, *P* < 0.0001) and whether the patient had been exposed to MOPP (RR 3.61, 95% CI 1.64–7.95, *P* = 0.0009) or lomustine chemotherapy (RR 4.53, 95% CI 1.96–10.44, *P* = 0.001). Following adjustment for these factors in the multivariate model the relative risk associated with transplantation was 1.83 (95% CI 0.66–5.11, *P* = 0.25). This study provides no evidence of a significantly increased risk of MDS/AML associated with BEAM therapy and autologous transplantation in Hodgkin's disease. Concern over MDS/AML should not mitigate against the timely use of this treatment modality. © 1999 Cancer Research Campaign


*British Journal of Cancer* (1999) **81**(3), 476–483
© 1999 Cancer Research Campaign
Article no. bjoc.1999.0718

### High-dose BEAM chemotherapy with autologous haemopoietic stem cell transplantation for Hodgkin's disease is unlikely to be associated with a major increased risk of secondary MDS/AML

**CN Harrison[1], W Gregory[1], G Vaughan Hudson[1], S Devereux[1], AH Goldstone[1], B Hancock[2], D Winfield[3], AK MacMillan[4], P Hoskin[4], AC Newland[5], D Milligan[6] and DC Linch[1]**

[1]University College Medical School, London, UK; [2]Weston Park Hospital, Whitham Rd, Sheffield, UK; [3]Royal Hallamshire Hospital, Glossop Rd, Sheffield, UK; [4]Mount Vernon Hospital, Rickmansworth Road, Northwood, UK; [5]Royal London Hospital, Whitechapel, London, UK; [6]Birmingham Heartands Hospital, Bordesby Green East, Birmingham, UK

**Summary** Hodgkin's disease is curable in the majority of patients, although a proportion of patients are resistant to or relapse after initial therapy. High-dose therapy with autologous stem cell support has become the standard salvage therapy for patients failing chemotherapy, but there have been reports of a high incidence of myelodysplasia/acute myeloid leukaemia (MDS/AML) following such treatment. Patients who receive such therapy form a selected group, however, who have already been subjected to other leukaemogenic factors, such as treatment with alkylating agents. In order to ascertain the true risk of MDS/AML, comparison must be made with other patients subjected to the same risks but not undergoing transplantation. We report a retrospective comparative study of 4576 patients with Hodgkin's disease from the BNLI and UCLH Hodgkin's databases, which includes 595 patients who have received a transplant. Statistical analysis including Cox's proportional hazards multivariate regression model with time-dependent covariates was employed. This analysis reveals that the risk of developing MDS/AML was dominated by three factors, namely quantity of prior therapy (relative risk [RR] 2.01, 95% confidence intervals [CI] 1.49–2.71, for each treatment block, $P < 0.0001$) and whether the patient had been exposed to MOPP (RR 3.61, 95% CI 1.64–7.95, $P = 0.0009$) or lomustine chemotherapy (RR 4.53, 95% CI 1.96–10.44, $P = 0.001$). Following adjustment for these factors in the multivariate model the relative risk associated with transplantation was 1.83 (95% CI 0.66–5.11, $P = 0.25$). This study provides no evidence of a significantly increased risk of MDS/AML associated with BEAM therapy and autologous transplantation in Hodgkin's disease. Concern over MDS/AML should not mitigate against the timely use of this treatment modality. © 1999 Cancer Research Campaign

**Keywords:** secondary myeloid malignancy; Hodgkin's disease; transplantation

High-dose therapy with autologous bone marrow (ABMT) or peripheral blood stem cell (PBSC) support is increasingly used in the treatment of patients with poor prognosis Hodgkin's disease (Jagannath et al, 1989; Wheeler et al, 1990; Reece et al, 1991; Chopra et al, 1993; Linch et al, 1993). Initial studies largely focused on the treatment of patients with primary refractory or multiply relapsed Hodgkin's disease, but, as this modality of therapy became more widely accepted, it has frequently been used at the time of first relapse. In some centres, patients deemed to be at high risk of relapse are being transplanted in first remission (Carella et al, 1991).

Concern about the increased use of high-dose therapy has recently been raised, however, by a number of reports containing details of patients with Hodgkin's disease and non-Hodgkin's lymphoma who have developed myelodysplasia (MDS) or acute myeloid leukaemia (AML) after autologous transplantation (Marrolleau et al, 1993; Darrington et al, 1994; Miller et al, 1994; Stone et al, 1994). Most of these studies have not included patients with uniform diagnoses and comparisons with similar patients not receiving high-dose therapy have not been made. This latter point is of great importance as secondary leukaemia is a well documented complication of standard dose chemotherapy for lymphoma, especially Hodgkin's disease (Tucker et al, 1988; Devereux et al, 1990; Swerdlow et al, 1993). A number of risk factors have been identified in the literature. The most important is the use of combination chemotherapy regimens containing alkylating agents (e.g. MOPP: mustine, vincristine, procarbazine, prednisolone), and the cumulative total dose of such drugs (Pedersen-Bjergaard et al, 1987; Devereux et al, 1990). Maintenance treatment with chlorambucil (Glicksman et al, 1982), or lomustine (CCNU) (Devereux et al, 1990) are associated with a particularly high risk of secondary leukaemia. Other factors identified in some, but not all, series include the use of combined modality therapy, advanced age and stage at diagnosis, and previous splenectomy. There may also be an increased risk intrinsic to Hodgkin's disease itself as sporadic cases occurring in patients untreated for Hodgkin's disease have been reported (Lacher and Susman, 1963).

Previously we have attempted a matched pair analysis of Hodgkin's patients who have undergone high-dose therapy with the BEAM (BCNU (carmustine), Etoposide, Ara-C (cytarabine) and Melphalan) regimen and stem cell transplantation with

*Received 14 December 1998*
*Revised 1 April 1999*
*Accepted 12 April 1999*

*Correspondence to:* DC Linch, Department of Haematology, University College Medical School, 98 Chenies Mews, London WC1E 6HX, UK

**Table 1** Characteristics of all 4576 patients with Hodgkin's disease and of 595 patients with Hodgkin's disease who received a transplant

| | | All patients | | Transplant patients | |
|---|---|---|---|---|---|
| | | *n* | % | *n* | % |
| Sex | M | 2824 | (61.7%) | 386 | (64.9%) |
| | F | 1752 | (38.3%) | 209 | (35.1%) |
| Age at diagnosis (years) | 0–15 | 74 | (1.6) | 20 | (3.4) |
| | 16–25 | 1442 | (31.5) | 239 | (40.2) |
| | 26–35 | 1237 | (27) | 218 | (36.6) |
| | 36–45 | 719 | (15.7) | 92 | (15.5) |
| | 46–55 | 499 | (10.9) | 25 | (4.2) |
| | 56–65 | 411 | (9) | 1 | (0.1) |
| | 66–75 | 177 | (3.9) | | |
| | 76–85 | 17 | (0.4) | | |
| | | mean 35.09 years, SD 14.87 (range 7–80 years) | | mean 27.85 years, SD 8.57 (range 7–56 years) | |
| Stage | ? | 6 | (0.2) | 4 | (0.7) |
| | 1 | 963 | (21.0) | 41 | (6.9) |
| | 2 | 1561 | (34.1) | 207 | (34.8) |
| | 3 | 1196 | (26.1) | 202 | (33.9) |
| | 4 | 850 | (18.6) | 141 | (23.7) |
| B symptoms | ? | 10 | (0.2) | 4 | (0.7) |
| | Y | 1818 | (39.7) | 243 | (40.8) |
| | N | 2748 | (60.1) | 348 | (58.5) |
| Splenectomy | Y | 1263 | (27.6) | 55 | (9.2) |
| | N | 3313 | (72.4) | 540 | (90.8) |
| Treatment blocks | 1 | 2336 | (51) | 1 | (0.1) |
| | 2 | 1115 | (24.4) | 135 | (22.7) |
| | 3 | 680 | (14.9) | 232 | (39.0) |
| | 4 | 356 | (7.8) | 154 | (25.9) |
| | >5 | 89 | (1.9) | 73 | (12.3) |
| MOPP | Y | 1139 | (24.9) | 146 | (24.5) |
| | N | 3437 | (75.1) | 449 | (75.5) |
| CCNU | Y | 147 | (3.2) | 16 | (2.7) |
| | N | 4429 | (96.8) | 579 | (97.3) |
| ABMT conditioning | BEAM | | | 582 | (97.8) |
| | Other | | | 13 | (2.2) |
| Source of stem cells | BM | | | 366 | (61.5) |
| | PBSC | | | 208 | (35) |
| | Both | | | 11 | (1.8) |
| | ? | | | 10 | (1.7) |

Of the 13 patients who received other conditioning a sole patient received each of NAM (neomycin, adriamycin and melphalan), allogeneic transplant, cyclophosphamide/TBI, melphalan alone, and a single patient received cyclophosphamide, BCNU and vincristine, two patients received BEM (BCNU, etoposide and melphalan), and two patients were conditioned with melphalan and etoposide. In four patients the conditioning regime was unknown. Nine patients underwent two transplantation procedures. Abbreviations: BM = bone marrow, PBSC = peripheral blood stem cells including CD34+ selected cells.

non-transplanted patients for factors known to increase the risk of secondary myeloid malignancy (Harrison et al, 1996). This suggested that there was no significant increase in risk of MDS/AML with BEAM but the confidence limits were large and the study was limited by the highly selected nature of the transplant patients causing difficulty in identifying suitable controls, even from a large Hodgkin's disease database.

In the current study we have therefore retrospectively analysed a larger cohort of 4576 patients with Hodgkin's disease of whom 595 had received high-dose therapy and an autologous bone marrow or peripheral blood stem cell transplant. The incidence of MDS/AML was determined in the series as a whole and after a transplant procedure. The factors increasing the risk of developing a secondary myeloid malignancy were identified and using sophisticated statistical modelling to account for time and accrual of risk factors we have been able to distinguish between the leukaemogenic effects of the high-dose chemotherapy and other factors such as previous and alternative conventional dose therapies.

### PATIENTS AND METHODS

### Patients

This analysis was performed upon 4576 patients with Hodgkin's disease composed of 4347 patients registered on the British National Lymphoma Investigation (BNLI) database, and an additional 229 sequential patients, not previously registered with the BNLI who were treated with BEAM chemotherapy and ABMT or PBSCT at UCLH between September 1983 and May 1995. A total of 595 patients were transplanted, 336 at UCLH. The BNLI is a long-established, large clinical collaborative trials group with follow-up of patients documented on a 6-monthly basis for the

© 1999 Cancer Research Campaign

**Table 2** Factors considered in the univariate analysis

| Variable | $\chi^2$ | *P*-value | Relative risk | 95% CI |
|---|---|---|---|---|
| Number of treatments | 44.52 | < 0.001 | 2.62 | 2.02–3.41 |
| MOPP chemotherapy | 29.96 | < 0.001 | 7.05 | 3.36–14.83 |
| CCNU therapy | 30.49 | < 0.001 | 16.04 | 7.47–34.45 |
| Alkylating agent | 26.31 | < 0.001 | 21.87 | 2.99–159.9 |
| Transplant | 12.17 | 0.0005 | 5.35 | 2.38–12.00 |
| Gender | 2.98 | 0.0842 | 1.81 | 0.92–3.55 |
| Age at each block | 2.49 | 0.1148 | 1.02 | 1.00–1.04 |
| Year of diagnosis | 0.36 | 0.5485 | 1.02 | 0.96–1.08 |
| Age at diagnosis | 0.34 | 0.5579 | 1.02 | 1.00–1.05 |
| Splenectomy | 0.08 | 0.7803 | 1.04 | 0.51–2.12 |

Relative risk depends upon the number of categories for that variable, e.g. relative risk for number of treatments relates to adjacent treatment thus comparing one block versus four blocks the relative risk is $2.62^3$ or 18.

**Table 3** Factors considered in the multivariate analysis

| Variable | $\chi^2$ | *P*-value | Relative risk | 95% CI |
|---|---|---|---|---|
| Significant variables entered into the multivariate model | | | | |
| Number of treatments | 18.58 | < 0.0001 | 2.01 | 1.49–2.71 |
| MOPP chemotherapy | 11.09 | 0.0009 | 3.61 | 1.64–7.95 |
| CCNU therapy | 10.38 | 0.0013 | 4.53 | 1.96–10.44 |
| Other non-significant variables considered | | | | |
| Gender | 4.47 | 0.0345 | 2.08 | 1.06–4.09 |
| Year of diagnosis | 3.83 | 0.0503 | 1.07 | 1.00–1.15 |
| Age at each block | 3.75 | 0.0528 | 1.03 | 1.00–1.05 |
| Alkylating agent | 3.21 | 0.0732 | 4.97 | 0.61–40.78 |
| Age at diagnosis | 2.92 | 0.0875 | 1.02 | 1.00–1.05 |
| Transplant | 1.30 | 0.2542 | 1.83 | 0.66–5.11 |
| Splenectomy | 0.01 | 0.9203 | 1.04 | 0.51–2.12 |

first 5 years from diagnosis and at yearly intervals thereafter, unless relapse occurs, in which case follow-up reverts to the more frequent schedule. Similar regular updating is performed on the UCLH transplant database. The proportion of patients on the BNLI database lost to follow-up is approximately 2% (Swerdlow et al, 1993), and of the 336 UCLH transplanted patients only four have been lost to follow-up, three of whom reside permanently overseas.

Criteria for transplantation at UCLH and within the BNLI trials were until recently as previously described (Khwaja et al, 1992; Chopra et al, 1993; Linch et al, 1993). They were:

1. A poor prognosis disease failing to achieve a remission on MOPP-like chemotherapy
2. failure to achieve complete remission or relapsing within 1 year of an initial alternating regimen (e.g. LOPP/EVAP: chlorambucil, vincristine, procarbazine, prednisolone/etoposide, vinblastine, doxorubicin, and prednisolone)
3. those patients who had failed on two or more lines of chemotherapy.

More recently some patients have been transplanted after relapsing beyond 1 year after obtaining a complete remission (CR) on alternating therapy. Only one patient in this series was transplanted in first CR.

Since number and type of treatment received are important predictors of the outcome that we were assessing (secondary MDS/AML), all treatments given over the 28 years of patient accrual were included in the analysis. Information was available on a total of 8500 treatment ‘blocks’. Table 1 contains clinical details pertaining to the 4576 patients on the combined database and the 595 who underwent transplantation respectively.

### High-dose therapy regimen

Altogether 582 (98%) of the transplanted patients received BEAM. The BEAM regimen has been previously described and contains BCNU (carmustine) 300 mg $m^{-2}$ day 1, etoposide 200 mg $m^{-2}$ days 2–5, Ara-c (cytarabine) 200 mg $m^{-2}$ twice daily on days 2–5 and melphalan 140 mg $m^{-2}$ on day 6. During the time of this study various doses of etoposide were used as part of a dose escalation study at UCLH (Mills et al, 1995), six patients received 100 mg $m^{-2}$, 277 received 200 mg $m^{-2}$, one received 300 mg $m^{-2}$, 41 had 400 mg $m^{-2}$ and 11 had 600 mg $m^{-2}$. A further 13 patients received other high-dose chemotherapy regimens and two received regimens containing total body irradiation.

As a source of stem cells 366 (61.5%) of the transplanted patients received ABMT, 208 (35%) PBSC (including six CD34+ selected transplants), and 11 (1.8%) ABMT and PBSC. In ten (1.7%) patients the source of stem cells is unclear.

### METHODS

For the purposes of the analysis the patients’ treatment was analysed as an individual block so, for example, a patient who was initially treated with mantle radiation then relapsed and was given six courses of LOPP/EVAP would have been subject to two treatment

 © 1999 Cancer Research Campaign

**Table 4** Characteristics of 35 patients with Hodgkin's disease who developed MDS/AML

| | ***n*** | **%** | |
|---|---|---|---|
| Sex | Male | 17 | (48.6%) |
| | Female | 18 | (51.4) |
| Age at diagnosis of | 16–25 | 16 | (45.7) |
| Hodgkin's disease | 26–35 | 5 | (14.3) |
| | 36–45 | 5 | (14.3) |
| | 46–55 | 2 | (5.7) |
| | 56–65 | 5 | (14.3) |
| | 66–75 | 2 | (5.7) |
| | mean 35.11 years, SD 17.09, (range 16–72 years) | | |
| Stage | 1 | 2 | (5.7) |
| | 2 | 14 | (40.0) |
| | 3 | 12 | (34.3) |
| | 4 | 7 | (20) |
| B symptoms | Y | 16 | (45.7) |
| | N | 19 | (54.3) |
| Splenectomy | Y | 15 | (42.9) |
| | N | 20 | (57.1) |
| Treatment blocks | 1 | 9 | (25.75) |
| | 2 | 7 | (20.0) |
| | 3 | 9 | (25.75) |
| | 4 | 6 | (17.1) |
| | >5 | 4 | (11.4) |
| MOPP | Y | 24 | (68.6) |
| | N | 11 | (31.4) |
| CCNU | Y | 9 | (25.7) |
| | N | 26 | (74.3) |
| BEAM | Y | 8 | (22.9) |
| | N | 27 | (77.1) |
| Stem cell source in | ABMT | 5 | (62.5) |
| BEAM recipients | PBSC | 2 | (25) |
| Both | 1 | (12.5) | |

A single patient was excluded from the subsequent analysis as the diagnosis of MDS was made at the same time as that of the original Hodgkin's disease.

blocks first the radiotherapy, and secondly the LOPP/EVAP chemotherapy. Each 'block' of treatment was considered as a potential contributing factor to the development of MDS/AML, in terms of the treatment given in that block, the number of blocks already given, the age at the start of that block, and the time since the first treatment for Hodgkin's disease. To simplify graphical presentation of these results the times between the start of each block and either the end of that block, occurrence of AML, or death were calculated for each block. Occurrence of MDS/AML was considered an event, the other two end points were considered censored. These event times were then plotted and analysed using actuarial methods. The Figures shown therefore demonstrate the incidence of MDS/AML from the time of diagnosis or the start of a new treatment block. Thus for the purposes of the graphs which plot incidence of MDS/AML from the start of each treatment block, if the patient is receiving, or has received, the treatment under consideration by the start of that block, their MDS/AML risk is assumed to be related to that treatment. As an example, consider a patient received radiotherapy for early-stage disease, relapsed 5 years later and was given MOPP, relapsed again 4 years after this and received a transplant, relapsed again 3 years later and received LOPP/EVAP, and finally contracted AML 2 years after this. There are four treatment blocks for this patient. When comparing MOPP versus other treatments, the first block (5 years) will be considered in the 'other' category, and the last three blocks (4, 3 and 2 years respectively) will be included in the MOPP group. When comparing transplants versus other treatments, the first two treatment blocks will be considered in the 'other' category, and the last two will be included in the transplant group. The first three times will be censored, since the patient did not get MDS/AML up to that time. The last time will be complete.

A multivariate approach was then employed to investigate whether the incidence of MDS/AML in the transplanted patients was different from that in the non-transplanted patients, allowing for other significant predictors. Some of these predictors were already known (e.g. the number of previous treatment blocks, prior use of CCNU) others were checked specifically for this analysis because of information from previous reports or *a priori* reasoning. The complete list of factors considered is given in Tables 2 and 3.

### Statistical analysis

Actuarial incidence rates of MDS/AML were calculated using the method of Kaplan and Meier (1958) with significance determined using the log-rank test (Peto et al, 1977).

Incidence rates adjusted for other factors, along with adjusted log-rank statistics, were calculated using the method described by Gregory (1988). Thus, for example, the incidence of MDS/AML in transplants versus non-transplants can be plotted as it would have looked if there had been an equal proportion of patients

*© 1999 Cancer Research Campaign* 

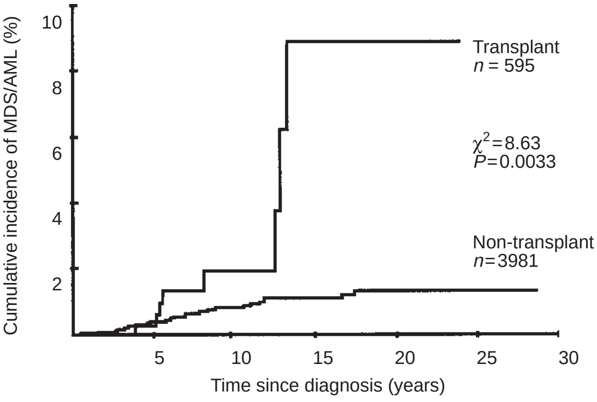

**Figure 1** The cumulative incidence of MDS/AML from the time of diagnosis in 595 patients with Hodgkin's disease undergoing transplantation, and 3981 patients who did not undergo transplantation

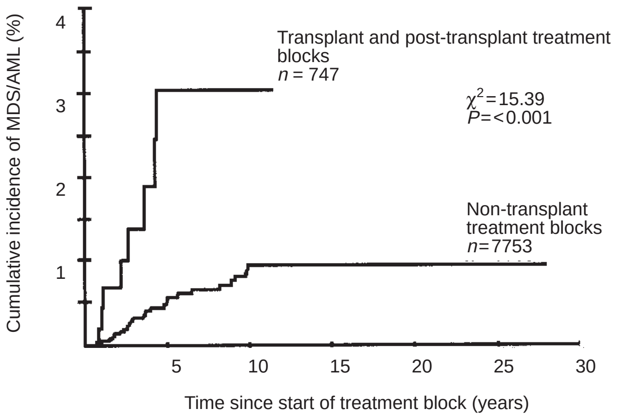

**Figure 2** The cumulative incidence of MDS/AML in transplant and non-transplant patients shown since the start of each treatment block. There are 747 transplant treatment blocks representing 595 transplants, and 152 blocks of post-transplant treatment having each number of prior treatments in the two groups (transplants and non-transplants). This comparison is actually shown in Figure 3.

Cox's proportional hazards multivariate regression model with time-dependent covariates (Cox, 1972) was used to investigate whether a transplant at any time increased the risk of MDS/AML. Other time-dependent covariates such as the administration of MOPP chemotherapy were also included in the model. Year of treatment was included to ensure that changes in patient management over the long periods of evaluation was not a relevant factor. A forward step-wise method was used to choose significant variables, with a cut-off *P*-value of 0.01. This value was chosen because of the large number of factors considered in the analysis.

### RESULTS

Thirty-five patients developed MDS/AML from the total cohort of 4576, and clinical details pertaining to these patients are shown in Table 4. In one case the diagnosis of MDS was made at the same time as that of Hodgkin's disease, and the MDS was thus considered to be a co-existing condition in this patient and could not be therapy related. This patient was therefore censored from further analyses.

A relatively high proportion (22.9%) of the patients who developed MDS/AML had undergone transplantation as part of the treatment for their Hodgkin's disease, whereas the proportion of patients in this total series who had received high-dose therapy was 13%. The actuarial incidence of MDS/AML in the 595 patients whom received a transplant was 0.25%, 1.9% and 9.0% at 5, 10 and 20 years respectively from the time of diagnosis. In the 595 transplanted patients the actuarial incidence from the time of transplant was 3.1% at 5 years (95% CI 1.5–6.3%). In the untransplanted patients the actuarial incidence of MDS/AML from the time of diagnosis was 0.4%, 0.8% and 1.3% at 5, 10 and 20 years respectively from the time of diagnosis. This unadjusted difference in the actuarial incidence of MDS/AML from the time of diagnosis between transplanted and non-transplanted patients is significant and is shown in Figure 1. Of the patients who had an autograft and developed MDS/AML, all eight had BEAM conditioning and five received ABMT alone, two PBSC and one both sources of stem cells.

It is also notable that compared to the patient group as a whole a much higher proportion of patients developing MDS/AML had

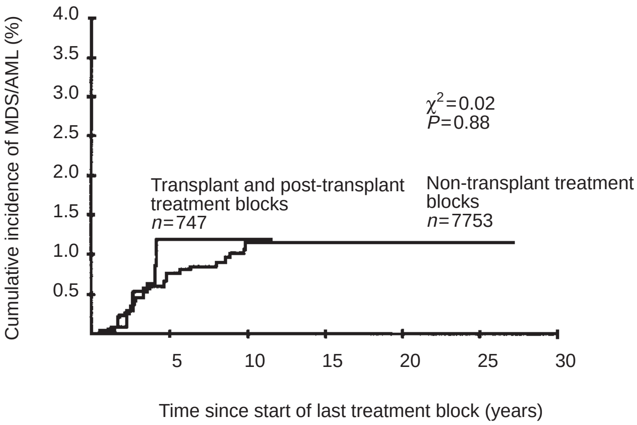

**Figure 3** The cumulative incidence of MDS/AML in transplant and non-transplant patients shown since the start of each treatment block and adjusted for factors identified from the multivariate analysis. There are 747 transplant treatment blocks representing 595 transplants, and 152 blocks of post-transplant treatment

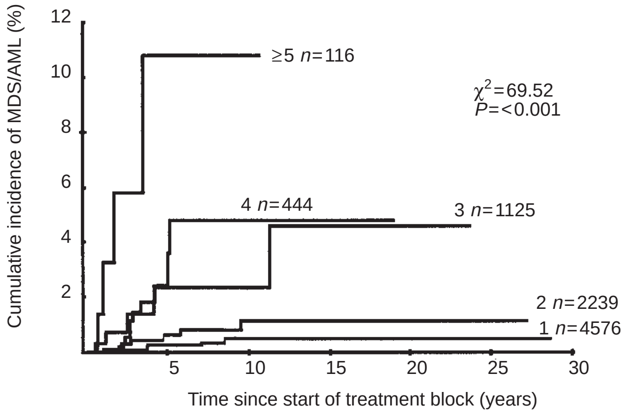

**Figure 4** The influence of number of treatment blocks upon the cumulative incidence of MDS/AML in 4576 Hodgkin's disease patients shown since the start of each treatment block

© 1999 Cancer Research Campaign

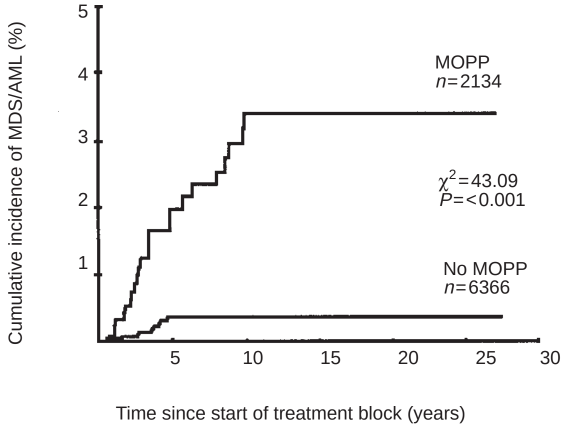

**Figure 5** The influence of MOPP chemotherapy upon the cumulative incidence of MDS/AML in 4576 Hodgkin's disease patients shown since the start of each treatment block

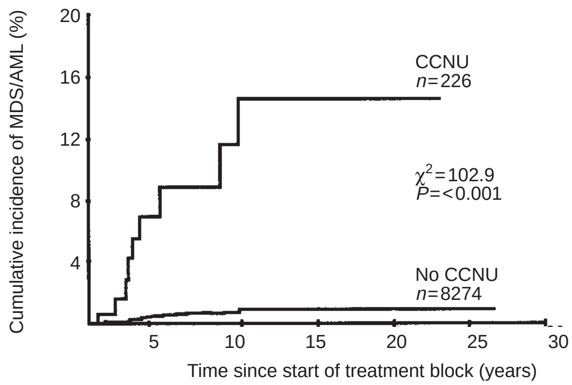

**Figure 6** The influence of lomustine (CCNU) chemotherapy upon the cumulative incidence of MDS/AML in 4576 Hodgkin's disease patients shown since the start of each treatment block received MOPP (68.6% (MDS/AML) vs 24.9% for the total 4576 patients who had received MOPP), or CCNU chemotherapy (25.7% (MDS/AML) vs 3.2%). Furthermore, 54.3% of the patients developing MDS/AML had received three or more treatment blocks compared to 24.6% of the total group (see Tables 1 and 4). Similarly, the patients who received a transplant were also different from the total series of 4576 patients with Hodgkin's disease with 24.5% and 2.7% receiving MOPP chemotherapy and CCNU chemotherapy respectively, and 77.2% having three or more treatment blocks. Importantly, each of these factors has previously been demonstrated to result in a significantly increased risk of secondary myeloid malignancy in Hodgkin's disease. Therefore a Cox's proportional hazards multivariate regression model with time-dependent covariates including the use of transplants, MOPP and CCNU was utilized to investigate whether a transplant at any time increased the risk of MDS/AML independently of other such risk factors.

Factors considered in the univariate analysis (taken from the Cox model without any covariates in order to make it as comparable as possible with the multivariate analysis), the *P*-value assigned to them, and their relative risk are shown in Table 2.

The multivariate model with time dependent variates was employed to assess the influence of transplantation on risk of MDS/AML. The factors analysed and the results of the multivariate analysis are shown in Table 3. Transplantation is not associated with a significant risk of secondary myeloid malignancy, $P = 0.25$, relative risk 1.83 (95% CI 0.66–5.11). The change from transplant being highly significant in the univariate analysis to being not significant in the multivariate analysis occurred immediately upon the inclusion of 'number of treatment blocks' in the Cox model.

To show this result graphically the risk of MDS/AML in the 595 patients who received a transplant was adjusted for the number of treatment blocks given and plotted from the start of each treatment block as detailed in the Methods section (Figures 2 and 3). For the transplant patients the number of treatment blocks is 747, reflecting the transplant and any subsequent treatment blocks. There was no significant difference in incidence of MDS/AML in the transplanted and non-transplanted groups after this adjustment was made.

Risk factors for the development of MDS/AML identified from the multivariate analysis are therefore number of treatment attempts or blocks, use of MOPP chemotherapy and CCNU chemotherapy. The effects of these three factors can be seen in Figures 4, 5 and 6 with relative risks of 2.01 (95% CI 1.49–2.71) for each treatment block, 3.61 (95% CI 1.64–7.95) for the use of MOPP and 4.53 (95% CI 1.96–10.44) for the use of CCNU.

None of the other variables remained significant. It is noteworthy that there was no apparent increased risk associated with the use of alkylating agents, but this is probably because of the increased risk ascribed to MOPP as well as the number of treatment blocks. It was clear in the multivariate analysis that MOPP chemotherapy was a considerably more significant risk factor than alkylating agent therapy, even though they are correlated. To demonstrate this point after inclusion of the number of treatment blocks the $\chi^2$ and *P*-values for MOPP and alkylating agents were 14.25 $P = 0.0002$ and 7.83 $P = 0.005$ respectively.

### DISCUSSION

In this total series of 4576 patients the actuarial incidence of MDS/AML was 0.4% and 0.9% at 5 and 10 years respectively, which is similar to the incidence reported from previous studies of the BNLI data base (Devereux et al, 1990; Swerdlow et al, 1993) despite the inclusion of an additional cohort of 229 transplanted patients from the UCLH transplant database. The incidence of secondary MDS/AML is undoubtedly increased in the cohort of Hodgkin's disease patients undergoing high-dose therapy and autologous transplantation compared to those patients who did not receive high-dose therapy. This is in accord with reports from several other series of lymphoma patients in which there is a projected cumulative incidence of MDS/AML at 5 years post-high-dose therapy of 6–24% (Marrolleau et al, 1993; Darrington et al, 1994; Miller et al, 1994; Stone et al, 1994, Traweek et al, 1994). Specifically, among Hodgkin's disease patients in the Nebraska series the risk in those patients still alive at 5 years was estimated at 11% (Darrington et al, 1994) and in the Minneapolis series 15% (Miller et al, 1994). These incidences are higher than in the UK series reported here, where the incidence at 5 years from transplantation was 3.1% (95% CI 1.5–6.3%).

It is possible that the apparent difference in incidence rates between our own study and those of other groups relates to patient selection for high-dose therapy procedures or possibly to the

© 1999 Cancer Research Campaign specific high-dose therapy regimen. The large majority of patients in our series were conditioned with BEAM chemotherapy (98%) with very few receiving total body irradiation (0.3%), and in the Nebraska series the use of TBI was identified as increasing the risk of secondary MDS/AML (Darrington et al, 1994). The diagnosis of MDS may be difficult in the post-transplant period as the possibility of disordered haematopoietic reconstitution may itself give rise to dysplastic features. In such cases it is the practice at UCLH to carry out cytogenetic examination of the bone marrow and X-linked chromosome inactivation patterns in females to search for clonal abnormalities (Gale et al, 1996). However, as the natural history of therapy related or secondary MDS is usually one of rapid evolution to bone marrow failure or frank leukaemia (Devereux, 1991), delayed rather than mistaken diagnosis is a more likely occurrence.

The crucial issue is whether the occurrence of MDS/AML post-transplantation is due to the high-dose therapy per se or is merely indicative of the fact that the transplanted patients have an increased risk due to their previous, often extensive, therapy. Secondary MDS/AML in Hodgkin’s disease is a very well described phenomenon and analyses of its occurrence, natural history and aetiology have been reported from many institutions (Tucker et al, 1988; Devereux et al, 1990; Pedersen-Bjergaard and Larsen, 1982; Pedersen-Bjergaard et al, 1987). Pre-eminent amongst risk factors identified are the use of combination chemotherapy regimes containing alkylating agents (e.g. MOPP), and compounding risk with successive number of attempts at treatment, particularly when that number exceeds two (Devereux et al, 1990). In this series 99.9% of the transplanted patients received two or more, and 77.2% three or greater treatment blocks of therapy, so a high risk of developing MDS/AML is inevitable.

Previous studies have been unable to distinguish between the effects of high-dose therapy and previous therapy as the transplant series have been restricted to patients who received that modality of treatment. There has been circumstantial evidence that pre-transplant therapy is the most significant aetiological factor in that the majority of patients who develop MDS/AML have had multiple treatment attempts prior to high-dose therapy and chronically abused stem cells are most likely to undergo malignant transformation (Devereux et al, 1990). In addition, MDS/AML rarely occurs after allogeneic transplantation, and although mixed chimaeras do occasionally occur, this suggests that it is the infused marrow, not exposed to the high-dose therapy, rather than residual marrow that is involved in the leukaemogenic process (Stone, 1994).

A Danish series of 76 patients with both non-Hodgkin’s and Hodgkin’s lymphoma patients receiving BEAM and autologous stem cell support compared the cumulative risk of MDS/AML in patients with Hodgkin’s disease following increasing doses of alkylating agents and demonstrated a similar cumulative risk as from the time of first chemotherapy (Pedersen-Bjergaard et al, 1997). This study indirectly inferred that therapy with BEAM and stem cell support might not increase the risk of leukaemogenesis to any major degree. The case–control study we previously reported (Harrison et al, 1996) also suggested that treatment prior to transplantation was the major contributor to the myeloid malignancies described in the post-transplant period, but the analysis was restricted by the highly selected nature of transplant patients which limited the ability to identify suitable controls.

A case–control study (Andre et al, 1998) has been reported which matched Hodgkin’s disease patients treated with a transplant to three untransplanted controls for the following variables age at original diagnosis of Hodgkin’s disease, gender, stage, B symptoms and length of follow-up but not the quantity of prior treatment. In this study the 5-year cumulative incidence of MDS/AML in the transplanted patients was 4.3% (95% CI 1.9–9.3%), and the case–control analysis indicated that there was no significant increase in risk of MDS/AML following transplantation $P = 0.056$, relative risk 2.54 (95% CI 0.98–6.61). This study therefore also supports the large analysis reported here, even though controls were not selected according to their prior treatment.

In the current retrospective analysis we have been able to study 4576 patients with Hodgkin’s disease from the BNLI registry and UCLH transplant database who have detailed clinical and treatment records and well documented follow-up. In this series the majority of patients who developed MDS/AML post-transplantation had received ABMT. No conclusions can be drawn about the relative risks of patients receiving PBSC or ABMT, as the number of events is small and the follow-up of the PBSC recipients is markedly less than in the ABMT recipients. A comparison of the actuarial incidence of secondary myeloid malignancy in transplanted versus non-transplanted patients reveals an apparent increase in risk in the transplanted cohort. Closer examination of the information, however, combined with sophisticated statistical modelling demonstrated that the risk of MDS/AML was dominated by three factors, namely quantity of prior therapy and whether the patient had been exposed to MOPP chemotherapy or CCNU chemotherapy. It is important to note that the relative risk with number of treatments of 2.62 (2.02–3.41) applies to each block of therapy, and in multiply treated patients this becomes a major risk factor. Thus, for instance, the relative risk comparing one block versus four blocks is $2.62^3$ or 18. These factors should clearly be taken into account in all future analysis of the risks of secondary myeloid malignancy in Hodgkin’s disease, and for the first time it has been possible to show here that, once the influence of these risk factors was taken into account, there was no significantly increased risk attributable to the high-dose BEAM chemotherapy. It is not possible to say that there is absolutely no leukaemogenic effect of BEAM therapy, but merely there is no evidence of such an effect despite this very large study. Anxieties about leukaemogenesis should not therefore preclude the early use of such therapy which could reduce the use of more leukaemogenic alternative regimens.

### REFERENCES

Andre M, Henry-Amar M, Blaise D, Colombat P, Fleury J, Milpied N, Cahn J-Y, Pico J-L, Bastion Y, Kuentz M, Nedellec G, Attal M, Ferme C and Gisselbrecht C (1998) Treatment-related deaths and second cancer risk after autologous stem cell transplantation for Hodgkin’s disease. *Blood* **92**: 1933–1940

Carella AM, Carlier P, Congiu A, Occhini D, Nati S, Santini G, Pierluigi D, Giordano D, Bacigalupo A and Damasio E (1991) Autologous bone marrow transplantation as adjuvant treatment for high risk Hodgkin’s disease in first complete remission after MOPP/ABVD protocol. *Bone Marrow Transplant* **8**: 99–103

Chopra R, McMillan AK, Linch DC, Yuklea S, Taghipour G, Pearce R, Paterson K and Goldstone AH (1993) The place of high-dose BEAM therapy and autologous bone marrow transplantation in poor-risk Hodgkin’s disease. A single-centre eight-year study of 155 patients. *Blood* **81**: 1137–1145

Cox DR (1972) Regression models and life tables. *J Royal Stat Soc* (B) **34**: 187–220

Darrington DL, Vose JM, Anderson JR, Bierman PJ, Bishop MR, Chan WC, Morris ME, Reed EC, Sanger WG, Tarantolo SR, Weisenberger DD, Kessinger A and Armitage JO (1994) Incidence and characterisation of secondary myelodysplastic syndrome and acute myelogenous leukaemia following high dose chemoradiotherapy and autologous stem cell transplantation for lymphoid malignancies. *J Clin Oncol* **12**: 2527–2534

 © 1999 Cancer Research Campaign

Devereux S, Selassie TG, Vaughan Hudson G, Vaughan Hudson B and Linch DC (1990) Leukaemia complicating treatment for Hodgkin's disease: the experience of the British National Lymphoma Investigation. *Br Med J* **301**: 1077–1080

Devereux S (1991) Therapy-associated leukaemia. *Blood Rev* **5**: 138–145

Gale RE, Bunch C, Moir DJ, Patterson KP, Goldstone AH and Linch DC (1996) Demonstration of developing myelodysplasia/acute myeloid leukaemia in haematologically normal patients after high dose chemotherapy and autologous bone marrow transplantation using X-chromosome inactivation patterns. *Br J Haematol* **93**: 53–58

Glicksman AS, Pajak TF, Gottlieb A, Nissen N, Stutzman L and Cooper MR (1982) Second malignant neoplasms in patients successfully treated for Hodgkin's disease: a Cancer and Leukaemia Group B study. *Cancer Treat Rep* **66**: 1035–1044

Gregory W (1988) Adjusting survival curves for imbalances in prognostic factors. *Br J Cancer* **58**: 202–204

Harrison CN, Vaughan Hudson G, Perry A, Anderson L, MacMillan A, Devereux S, Goldstone AH and Linch DC (1996) BEAM and ABMT/PBSCT may reduce the risk of secondary leukaemia in Hodgkin's disease: a case control analysis with the BNLI database (Abstract). *Blood* **88**: 262a

Jagannath S, Armitage JO, Dicke KA, Tucker SL, Velasquez WS, Smith K, Vaughan WP, Kessinger A, Horwitz W, Hagemeister FB, McLaughlin P, Cabanillas F and Spitzer G (1989) Prognostic factors for response and survival after high dose cyclophosphamide, carmustine and etoposide with autologous bone marrow transplantation for relapsed Hodgkin's disease. *J Clin Oncol* **7**: 179–185

Kaplan EL and Meier P (1958) Nonparametric estimation from incomplete observations. *J Am Stat Assoc* **53**: 457–481

Khwaja A, Linch DC, Goldstone AH, Chopra R, Marcus RE, Wimperis JZ, Russell NH, Haynes AP, Milligan DW, Leyland MJ, Winfield DA, Hancock BW, Newland A, Durrant ST, Devereux S, Roitt S, Collins S and Vaughan Hudson G (1992) Recombinant human granulocyte-macrophage colony-stimulating factor after autologous bone marrow transplantation for malignant lymphoma: a British National Lymphoma Investigation double-blind, placebo-controlled trial. *Br J Haematol* **82**: 317–323

Lacher MJ and Susman LN (1963) Leukaemia and Hodgkin's disease. *Ann Intern Med* **59**: 369–378

Linch DC, Winfield D, Goldstone AH, Moir D, Hancock B, McMillan A, Chopra R, Milligan D and Vaughan Hudson G (1993) Dose intensification with autologous bone marrow transplantation in relapsed and resistant Hodgkin's disease: results of a BNLI randomised trial. *Lancet* **341**: 1051–1054

Marolleau JP, Brice P, Morel P and Gisselbrecht C (1993) Secondary acute myeloid leukaemia after bone marrow transplantation for malignant lymphomas (letter). *J Clin Oncol* **11**: 590–591

Miller JS, Arthur DC, Litz CE, Neglia JP, Miller WJ and Weisdorf DJ (1994) Myelodysplastic syndrome after autologous bone marrow transplantation: an additional late complication of curative cancer therapy. *Blood* **83**: 3780–3786

Mills W, Strang J, Goldstone AH and Linch DC (1995) Dose intensification of etoposide in the BEAM ABMT protocol for malignant lymphoma. *Leuk Lymphoma* **17**: 263–270

Pedersen-Bjergaard J and Larsen SO (1982) Incidence of acute nonlymphocytic leukaemia. Preleukaemia and acute myeloproliferative syndrome up to 10 years after treatment of Hodgkin's disease. *N Engl J Med* **307**: 965–971

Pedersen-Bjergaard-J, Specht L, Larsen SO, Ersboll J, Struck J, Hansen MM, Hansen HH and Nissen NI (1987) Risk of therapy-related leukaemia and preleukaemia after Hodgkin's disease: relation to age, cumulative dose of alkylating agents and time from chemotherapy. *Lancet* **II(1)**: 83–88

Pedersen-Bjergaard J, Pedersen M, Myhre J and Geisler C (1997) High risk of therapy-related leukaemia after BEAM chemotherapy and autologous stem cell transplantation for previously treated lymphomas is mainly related to primary chemotherapy and not to the BEAM-transplantation procedure. *Leukaemia* **11**: 1654–1660

Peto R, Pike MC, Armitage P, Breslow NE, Cox DR, Howard SV, Mantel N, McPherson K, Peto J and Smith PG (1977) Design and analysis of randomised clinical trials requiring prolonged observation of each patient. II. Analysis and examples. *Br J Cancer* **35**: 1–39

Reece DE, Barnett MJ, Connors JM, Fairey RN, Fay JW, Greer JP, Herzig GP, Herzig RH, Klingemann HG, LeMaisre CF, O'Reilly SE, Shepherd JD, Spinelli JJ, Voss NJ, Wolff SN and Philips GL (1991) Intensive chemotherapy with cyclophosphamide, carmustine and etoposide followed by autologous bone marrow transplantation for relapsed Hodgkin's disease. *J Clin Oncol* **9**: 1871–1879

Stone RM (1994) Myelodysplastic syndrome after autologous transplantation for lymphoma: the price of progress? *Blood* **83**: 3437–3440

Stone RM, Neuberg D, Soiffer R, Takvorian T, Whelan M, Rabinowe S, Aster JC, Leavitt P, Mauch P, Freedman AS and Nadler LM (1994) Myelodysplastic syndrome following autologous bone marrow transplantation for non-Hodgkin's lymphoma. *J Clin Oncol* **12**: 2535–2542

Swerdlow AJ, Douglas AJ, Vaughan Hudson G, Vaughan Hudson B and MacLennan KA (1993) Risk of second primary cancer after Hodgkin's disease in patients in the British National Lymphoma Investigation: relationships to host factors, histology, and stage of Hodgkin's disease and splenectomy. *Br J Cancer* **68**: 1006–1011

Traweek ST, Slovak ML, Nademanee AP, Brynes RK, Niland JC and Forman SJ (1994) Clonal karyotypic haematopoietic cell abnormalities occurring after autologous bone marrow transplantation for Hodgkin's disease and non-Hodgkin's lymphoma. *Blood* **84**: 957–963

Tucker MA, Coleman CN, Cox RS, Varghese A and Rosenberg SA (1988) Risk of second cancers after treatment for Hodgkin's disease. *N Engl J Med* **318**: 76–81

Wheeler C, Antin JH, Churchill W, Come SE, Smith BR, Bubley GJ, Rosenthal DS, Rappaport JM, Ault KA, Schnipper LE and Eder JP (1990) Cyclophosphamide, carmustine and etoposide with autologous bone marrow transplantation in refractory Hodgkin's disease and non-Hodgkin's lymphoma: a dose finding study. *J Clin Oncol* **8**: 648–656

*© 1999 Cancer Research Campaign*
